# Revised Criteria for Diagnosis and Staging of Alzheimer’s Disease

**DOI:** 10.1002/alz.095054

**Published:** 2025-01-09

**Authors:** Clifford R. Jack

**Affiliations:** ^1^ Mayo Clinic, Rochester, MN USA

## Abstract

The National Institute on Aging and the Alzheimer’s Association (NIA‐AA) convened three separate work groups in 2011 and a single work group in 2018 to create recommendations for the diagnosis and characterization of Alzheimer’s disease (AD). Several fundamental principles emerged from these efforts. These include, AD should be defined biologically, not by clinical syndromes. The disease is a continuum that is first evident with the appearance of brain pathologic changes in asymptomatic individuals and progresses through stages of increasing pathologic burden eventually leading to appearance/progression of clinical symptoms.

The Revised Criteria for Diagnosis and Staging of Alzheimer's Disease is the product of a work group sponsored by the Alzheimer’s Association. The revised Criteria update the 2018 Research Framework in response to major recent developments. First, since 2018, for the first time, treatments that target core disease pathology have received regulatory approval. Second, accepted biomarkers in 2018 were either CSF assays or imaging. Since then, plasma‐based biomarkers were developed, and some (but not all) demonstrate excellent diagnostic performance.

The objective of the work group was to propose objective criteria for diagnosis and staging AD to serve as a bridge between research and clinical care. These criteria are not intended to be specific clinical practise guidelines, but rather criteria for diagnosis and staging of AD that reflect current science.

Defining neurodegenerative diseases by their underlying biology rather than by non‐specific syndromic presentations has gained momentum in recent years. In addition, investigators increasingly recognize that across the spectrum of neurodegenerative disorders, the disease progresses along a continuum that begins with the appearance of the defining neuropathology while patients are asymptomatic. This symposium will examine the unifying theme of biologically based diagnosis and staging across neurodegenerative diseases.

